# The international PIACO study: pattern of surgical approaches for acute surgical pathologies in Spain *versus* UK. Was conservative treatment and open surgery during COVID-19 the way to go?

**DOI:** 10.1093/bjsopen/zrac089

**Published:** 2022-08-08

**Authors:** Hector Guadalajara, Marina Yiasemidou, José Luis Muñoz de Nova, Peter Sedman, Saul Fernandez Gonzalez, Sushil Maslekar, María Recarte Rico, Richard Egan, Luz Divina Juez, Kallingal Riyad, Javier García Septiem, Sonia Lockwood, Pablo Galindo Jara, Andrea Giorga, Mariana García Virosta, Julian Hance, Eduardo Lobo Martínez, Elena Martín-Pérez, Annabel Howitt, David Jayne, Ian Chetter, Damian García-Olmo, PIACO Collaboration Group, JM Fernández-Cebrián, JM Jover, D Acín-Gándara, E Perea-del-Pozo, S Dios-Barbeito, D Aparicio-Sánchez, VM Durán-Muñoz-Cruzado, Felipe Pareja-Ciuró, E Martin-Antona, O Cano-Valderrama, AJ Torres-Garcia, L Zarain-Obrador, M Durán-Poveda, Begoña Peinado-Iribar, D Fernandez-Luengas, I Pascual-Migueláñez, A Garcia-Chiloeches, A Puerta, E Martín-Pérez, Y García-del-Álamo-Hernández, R Maqueda-González, M Gutiérrez-Samaniego, L Colao-García, S Núñez-O’Sullivan, MA Vaquero, A Picardo-Nieto, A Blazquez-Martin, C Vera-Mansilla, S Soto-Schüte, A Gutiérrez-Calvo, J Mínguez-García, A Sanchez-Argüeso, S Hernández-Villafranca, S Qian-Zhang, S Gortazar-de-las-Casas, V Dominguez-Prieto, O Lopez-Fernandez, L Casalduero-García, MÁ Iparraguirre, M Florez-Gamarra, JM Argüello-de-Andrés, Benjamin Tallón-Iglesias, F Pereira-Perez, MÁ García-Ureña, Gloria Paeriro, ML Fuenmayor-Valera, R Pardo, M Pellen, M Basheer, R Harries, K Parkins, N Spencer, Z Li, J Burridge, H Wynn, M Mesri

**Affiliations:** Department of General and Digestive Surgery, Fundación Jimenez Díaz University Hospital, Madrid, Spain; Academic Clinical Lecturer, University of Hull, Hull, UK; Department of General and Digestive Surgery, La Princesa University Hospital, Instituto de Investigación Sanitaria Princesa (IIS-IP), Madrid, Spain; Upper Gi Surgery, Hull University Teaching Hospitals, Hull, UK; Department of General and Digestive Surgery, Fundación Jimenez Díaz University Hospital, Madrid, Spain; Colorectal Surgery, Leeds Teaching Hospitals, Leeds, UK; Department of General and Digestive Surgery, Tajo University Hospital, Madrid, Spain; Department of General Surgery, Swansea Bay UHB, Swansea University, Swansea, Wales, UK; Department of General and Digestive Surgery, Ramon y Cajal University Hospital, Madrid, Spain; Colorectal Surgery, Leeds Teaching Hospitals, Leeds, UK; Department of General and Digestive Surgery, La Princesa University Hospital, Instituto de Investigación Sanitaria Princesa (IIS-IP), Madrid, Spain; Colorectal Surgery, Bradford Teaching Hospitals, Bradford, UK; Department of General and Digestive Surgery, Torrejon University Hospital, Madrid, Spain; Colorectal Surgery, Leeds Teaching Hospitals, Leeds, UK; Department of General and Digestive Surgery, Infanta Sofia University Hospital, Madrid, Spain; Colorectal Surgery, Leeds Teaching Hospitals, Leeds, UK; Department of General and Digestive Surgery, Ramon y Cajal University Hospital, Madrid, Spain; Department of General and Digestive Surgery, La Princesa University Hospital, Instituto de Investigación Sanitaria Princesa (IIS-IP), Madrid, Spain; Department of Colorectal Surgery, Bradford Teaching Hospitals, Bradford, UK; Leeds Institute of Biomedical Sciences, University of Leeds, Leeds, UK; Academic Vascular Surgery, University of Hull, Hull, UK; Department of General and Digestive Surgery, Fundación Jimenez Díaz University Hospital, Madrid, Spain

*Dear Editor*

During the first wave of the SARS-CoV-2 pandemic, drastic restructuring of surgical services was applied, as part of a larger scheme aiming to protect health systems from being overwhelmed during the pandemic^1,2^. As a result of reserving resources to cope with the expected influx of patients with COVID-19, elective activity was postponed and acute surgical cases were treated preferably in an ambulatory rather than an inpatient setting^3–5^. Data during the early days of the pandemic, reporting worryingly high morbidity and mortality rates after surgery, led to acute cases being treated conservatively; surgery was reserved for severe cases or when conservative strategies had failed^5^.

A multicentre, comparative, international study was conducted in 16 centres in Spain and four in the UK, aiming to assess the impact of the COVID-19 pandemic and associated public health measures, on presentation and management of acute surgical pathologies.

Adult patients (older than 18 years) with a diagnosis of acute appendicitis, acute cholecystitis, acute diverticulitis, or perianal abscess (acute surgical inflammatory processes; ASIPs) during the national lockdown in each country (Spain, 14 March 2020 to 2 May 2020; UK, 23 March 2020 to 11 May 2020) were included. Patients were excluded if an ASIP had been diagnosed within 30 days before admission or if the admission was due to a scheduled surgery for definitive treatment of the ASIP. Patients with the above diagnoses within the same time frame the year before (Spain, 14 March 2019 to 2 May 2019; UK, 23 March 2019 to 11 May 2019) were historical controls.

Overall, 2181 cases were analysed (*Table 1*). A significantly lower percentage of mild cases of appendicitis presented during the pandemic in Spain and the UK, compared with the same time interval a year before (2020, 466 (57.7 per cent); 2019, 882 (65.2 per cent), *P* < 0.001; Supplementary material). Surgery was less frequently employed during the pandemic (509 (62.3 per cent) *versus* 921 (68 per cent), *P* = 0.007). When it was employed, it was significantly less frequently laparoscopic (251 (70.1 per cent) *versus* 622 (90.8 per cent), *P* < 0.001; *Table 1*).

**Table 1:** Patient features and ASIP treatment

	2020	2019	Total	*P*
**Patient characteristics**				
Age, (years) median (i.q.r.)	50 (34–67.2)	51 (36–67)	51 (35–67)	0.475
Male, *n* (%)	473 (57.9)	701 (51.4)	1174 (53.9)	0.003
CCI, median (i.q.r.)	1 (0–3)	1 (0–3)	1 (0–3)	0.192
**Treatment of ASIPs during lockdown**				
Appendicitis				
Overall procedures	287	509	796	<0.001
Laparoscopy	204	479	683	<0.001
Cholecystitis				
Overall procedures	50	144	194	<0.001
Laparoscopy	44	135	179	0.221
Diverticulitis				
Overall procedures	22	33	55	0.077
Laparoscopy	3	8	11	0.493
Perianal abscesses				
Overall procedures	150	235	385	0.646
Total				
Overall procedures	509	921	1430	0.007
Laparoscopy	251	622	873	<0.001

i.q.r., interquartile range; ASIP, acute surgical inflammatory process; CCI, Charlson co-morbidity index.

A correlation was demonstrated between the reduction in ASIP cases from 2019 to 2020 and the daily number of COVID-19 cases reported in Spain during the lockdown interval (*R* = 0.413, *P* = 0.030, *R*^2^ = 0.171), whereas, such a correlation was not demonstrated in the UK (*R* = 0.010, *P* = 0.944, *R*^2^ = 0.0001029; *Fig. 1*).

**Fig. 1:**
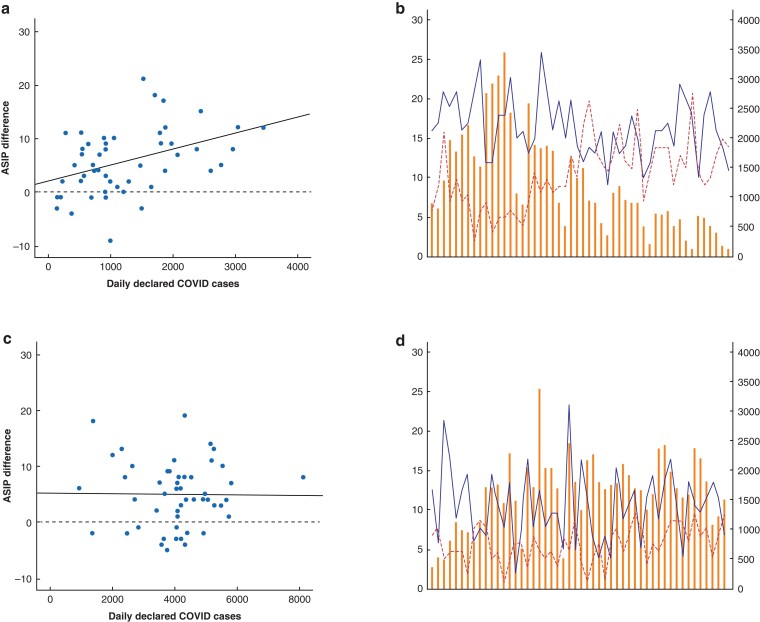
**a** Correlation of difference in number of acute surgical inflammatory process (ASIP) cases between 2019 and 2020 with daily COVID-19 cases in Spain. **b** Daily cases of COVID-19. Dotted line represents ASIP cases in 2020. Continuous line represents ASIPs in 2019 in Spain. **c** Correlation of difference in number of ASIP cases between 2019 and 2020 with daily COVID-19 cases in the UK. **d** Daily cases of COVID-19. Dotted line represents ASIP cases in 2020. Continuous line represents ASIPs in 2019 in the UK.

The independent predictors of non-surgical treatment of appendicitis and cholecystitis were presenting diagnosis in the UK (appendicitis OR 0.026, *P* < 0.001; cholecystitis, OR 0.148, *P* = 0.031), non-severe condition at diagnosis (appendicitis OR 11.433, *P* = 0.036; cholecystitis OR 7.944, *P* = 0.022) and a COVID-19-positive diagnosis (OR 0.142, *P* < 0.001). Predictors for open surgery were presenting in the UK (OR 0.152, *P* < 0.001) and COVID-19-positive status (OR 0.112, *P* = 0.002) (Supplementary material). There were no differences in mortality between the two years. Severity at diagnosis was the only independent predicting factor for major complications (OR 3.148, *P* = 0.003) (Supplementary material). Further information regarding patient and disease characteristics can be found in the Supplementary material. Subgroup analysis showed patients who tested positive in the UK were significantly older in age (53 *versus* 73 years, *P* = 0.002) and had significantly higher Charlson co-morbidity index (CCI) (1 *versus* 4, *P* < 0.001). No such differences were noticed in Spain (Supplementary material). Patients who tested positive for COVID-19 were more likely to have severe ASIPs at the time of diagnosis (71 *versus* 16, *P* = 0.002) (Supplementary material). Furthermore, they were less likely to be treated surgically (285 *versus* 19 patients, *P* = 0.002) and were more likely to have severe complications (33 *versus* 8 patients, *P* = 0.044) regardless of whether they were treated surgically or not.

This study demonstrates a shift towards conservative treatment and open surgery during the first wave of the COVID-19 pandemic. The single factor independently associated with severe complications (Clavien–Dindo classification) was severity of disease at presentation. Surgical treatment did not correlate with higher complication rates. These findings indicate that both surgical and conservative treatment had similar patient outcomes; hence, surgical treatment may have been as safe as conservative treatment during COVID-19.

Finally, a reduction of mild cases presenting in secondary care, was noted. This may indicate that cases of mild diverticulitis, cholecystitis, and appendicitis are currently being over-treated and can be successfully managed in the community instead.

## Collaborators

**PIACO Collaboration Group**

J.M Fernández-Cebrián (Department of General and Digestive Surgery, Ramon y Cajal University Hospital, Madrid, Spain); J.M. Jover (Department of General and Digestive Surgery, GetafeUniversity Hospital, Madrid, Spain); D. Acín-Gándara (Department of General and Digestive Surgery, Fuenlabrada University Hospital, Madrid, Spain). E. Perea-del-Pozo (Department of General and Digestive Surgery, Virgendel Rocio University Hospital, Sevilla, Spain); S. Dios-Barbeito (Department of General and Digestive Surgery, Virgendel Rocio University Hospital, Sevilla, Spain); D. Aparicio-Sánchez (Department of General and Digestive Surgery, Virgendel Rocio University Hospital, Sevilla, Spain); V.M. Durán-Muñoz-Cruzado (Department of General and Digestive Surgery, Virgen del Rocio University Hospital, Sevilla, Spain); Felipe Pareja-Ciuró (Department of General and Digestive Surgery, Virgen delRocio University Hospital, Sevilla, Spain); E. Martin-Antona (Department of General and Digestive Surgery, ClínicoSan Carlos University Hospital, Madrid, Spain); O. Cano-Valderrama (Department of General and Digestive Surgery, ClínicoSan Carlos University Hospital, Madrid, Spain); A.J Torres-Garcia (Department of General and Digestive Surgery, Clínico San Carlos University Hospital, Madrid, Spain); L. Zarain-Obrador (Department of General and Digestive Surgery, Rey JuanCarlos University Hospital, Madrid, Spain); M. Durán-Poveda (Department of General and Digestive Surgery, ReyJuan Carlos University Hospital, Madrid, Spain); Begoña Peinado-Iribar (Department of General and Digestive Surgery, QuironMadrid University Hospital, Madrid, Spain); D. Fernandez-Luengas (Department of General and Digestive Surgery, Quiron Madrid University Hospital, Madrid, Spain); I. Pascual-Migueláñez (Department of General and Digestive Surgery, LaPaz University Hospital, Madrid, Spain); A. Garcia-Chiloeches (Department of General and Digestive Surgery, Ramony Cajal University Hospital, Madrid, Spain); A. Puerta (Department of General and Digestive Surgery, Ramon y CajalUniversity Hospital, Madrid, Spain); E. Martín-Pérez (Department of General and Digestive Surgery, La PrincesaUniversity Hospital, Instituto de Investigación Sanitaria Princesa (IIS-IP), Madrid, Spain); Y. García-del-Álamo-Hernández (Department of General and Digestive Surgery, La Princesa University Hospital, Instituto de Investigación SanitariaPrincesa (IIS-IP), Madrid, Spain); R. Maqueda-González (Department of General and Digestive Surgery, LaPrincesa University Hospital, Instituto de Investigación Sanitaria Princesa (IIS-IP), Madrid, Spain); M. Gutiérrez-Samaniego (Department of General and Digestive Surgery, Torrejón University Hospital, Madrid, Spain); L. Colao-García (Department of General and Digestive Surgery, TorrejónUniversity Hospital, Madrid, Spain); S. Núñez-O’Sullivan (Department of General and Digestive Surgery, InfantaSofia University Hospital, Madrid, Spain); M.A Vaquero (Department of General and Digestive Surgery, InfantaSofia University Hospital, Madrid, Spain); A. Picardo-Nieto (Department of General and Digestive Surgery, InfantaSofia University Hospital, Madrid, Spain); A. Blazquez-Martin (Department of General and Digestive Surgery, Príncipede Asturias University Hospital, Madrid, Spain); C. Vera-Mansilla (Department of General and Digestive Surgery, Príncipede Asturias University Hospital, Madrid, Spain); S. Soto-Schüte (Department of General and Digestive Surgery, Príncipe deAsturias University Hospital, Madrid, Spain); A. Gutiérrez-Calvo (Department of General and Digestive Surgery, Príncipe de Asturias University Hospital, Madrid, Spain); J. Mínguez-García (Department of General and Digestive Surgery, Príncipede Asturias University Hospital, Madrid, Spain); A. Sanchez-Argüeso (Department of General and Digestive Surgery, Fundación Jiménez Díaz University Hospital, Madrid, Spain); S. Hernández-Villafranca (Department of General and Digestive Surgery, Fundación Jiménez Díaz University Hospital, Madrid, Spain); S. Qian-Zhang (Department of General and Digestive Surgery, FundaciónJiménez Díaz University Hospital, Madrid, Spain); S. Gortazar-de-las-Casas (Department of General and Digestive Surgery, Fundación Jiménez Díaz University Hospital, Madrid, Spain); V. Dominguez-Prieto (Department of General and Digestive Surgery, Fundación Jiménez Díaz University Hospital, Madrid, Spain); O. Lopez-Fernandez (Department of General and Digestive Surgery, Fundación Jiménez Díaz University Hospital, Madrid, Spain); L. Casalduero-García (Department of General and Digestive Surgery, Sanitas la Moraleja University Hospital, Madrid, Spain); M.Á Iparraguirre (Department of General and Digestive Surgery, Sanitas la Moraleja University Hospital, Madrid, Spain); M. Florez-Gamarra (Department of General and Digestive Surgery, Sanitasla Moraleja University Hospital, Madrid, Spain); J.M Argüello-de-Andrés (Department of General and Digestive Surgery, Sanitas la Moraleja University Hospital, Madrid, Spain); Benjamin Tallón-Iglesias (Department of General and Digestive Surgery, Sanitas la Moraleja University Hospital, Madrid, Spain); F. Pereira-Perez (Department of General and Digestive Surgery, Fuenlabrada University Hospital, Madrid, Spain); M.Á García-Ureña (Department of General and Digestive Surgery, Henares University Hospital, Madrid, Spain); Gloria Paeriro (Department of General and Digestive Surgery, Infanta LeonorUniversity Hospital, Madrid, Spain); M.L Fuenmayor-Valera (Department of General and Digestive Surgery, Infanta Leonor University Hospital, Madrid, Spain); R. Pardo (Department of General and Digestive Surgery, FundaciónJiménez Díaz University Hospital, Madrid, Spain); M. Pellen (Upper GI surgery, Hull University Teaching Hospitals, Hull, UK); M. Basheer (Colorectal Surgery, Mid Yorkshire NHS trust); R. Harries (Department of colorectal surgery, Swansea Bay University Health Board, Swansea, Wales, UK); K. Parkins (Department of colorectal surgery, Swansea Bay University Health Board, Swansea, Wales, UK); N. Spencer (Department of colorectal surgery, Swansea Bay University Health Board, Wales, UK); Z. Li (Department of colorectal surgery, Swansea Bay University Health Board, Wales, UK); J. Burridge (Department of colorectal surgery, Swansea Bay University Health Board, Wales, UK); H. Wynn (Colorectal surgery, Harrogate NHS Hospital, Harrogate, UK); M. Mesri (Academic Surgery, University of Hull, Hull, UK).

## Supplementary Material

zrac089_Supplementary_Data
