# Supplementary material for: The international PIACO study: pattern of surgical approaches for acute surgical pathologies in Spain versus UK. Was conservative treatment and open surgery during COVID-19 the way to go?
Source: BJS Open. 2022 Aug 8;6(4):zrac089. doi: 10.1093/bjsopen/zrac089 (PMC9359448; doi:10.1093/bjsopen/zrac089)
Supplement: zrac089_Supplementary_Data [file zrac089_supplementary_data.docx]

**The international PIACO Study: Pattern of surgical approaches for acute surgical pathologies in Spain vs. UK. Was conservative treatment and open surgery during COVID-19 the way to go?**

1. Hector Guadalajara, MD, PhD, Associate Professor of Surgery, Department of General and Digestive Surgery. Fundación Jimenez Díaz University Hospital. Madrid, Spain.
2. Marina Yiasemidou, PhD, NIHR Academic Clinical Fellow in General Surgery, University of Hull, ST8 colorectal surgery, Bradford Teaching Hospitals
3. José Luis Muñoz de Nova, MD, PhD Department of General and Digestive Surgery, La Princesa University Hospital, Instituto de Investigación Sanitaria Princesa (IIS-IP). Madrid, Spain
4. Peter Sedman, FRCS, Upper GI surgeon, Hull University Teaching Hospitals, Hull, UK
5. Saul Fernandez Gonzalez, Department of General and Digestive Surgery. Fundación Jimenez Díaz University Hospital. Madrid, Spain.
6. Sushil Maslekar, FRCS, Colorectal surgeon, Leeds Teaching Hospitals
7. Maria Recarte Rico, MD, Department of General and Digestive Surgery. Tajo University Hospital.
8. Richard Egan, FRCS, MD, Consultant Endocrine Surgeon, Department of General Surgery, Swansea Bay UHB, Honorary Senior Lecturer, Swansea University—
9. Luz Divina Juez, MD, Department of General and Digestive Surgery. Ramon y Cajal University Hospital. Madrid, Spain
10. Kallingal Riyad, FRCS, Consultant Colorectal Surgeon, Leeds Teaching Hospitals, Leeds, UK
11. Javier García Septiem, MD, PhD, Department of General and Digestive Surgery, La Princesa University Hospital, Instituto de Investigación Sanitaria Princesa (IIS-IP). Madrid, Spain
12. Sonia Lockwood, FRCS, Colorectal Consultant, Bradford Teaching Hospitals, Bradford, UK
13. Pablo Galindo Jara, MD, Department of General and Digestive Surgery. Torrejon University Hospital. Madrid, Spain
14. Andrea Giorga, MRCS IST Surgical trainee, Leeds teaching Hospitals, Leeds, UK
15. Mariana García Virosta, MD, Department of General and Digestive Surgery. Infanta Sofia University Hospital. Madrid, Spain
16. Julian Hance, FRCS, Colorectal Consultant, Leeds Teaching Hospitals
17. Eduardo Lobo Martínez, MD, PhD, Department of General and Digestive Surgery. Ramon y Cajal University Hospital. Madrid, Spain
18. Elena Martín-Pérez, MD, PhD, Department of General and Digestive Surgery, La Princesa University Hospital, Instituto de Investigación Sanitaria Princesa (IIS-IP). Madrid, Spain
19. Annabel Howitt, MBBS, Department of Colorectal Surgery, Bradford Teaching Hospitals, UK
20. David Jayne, FRCS Professor of Surgery, University of Leeds, Leeds, UK
21. Ian Chetter, FRCS, Professor of Surgery, University of Hull, Hull, UK
22. Damian García-Olmo, MD, PhD, Professor of Surgery, Department of General and Digestive Surgery. Fundación Jimenez Díaz University Hospital. Madrid, Spain
23. PIACO Collaboration Group†: Fernández-Cebrián José María, Blazquez Martin Agustín, Jover José María. Iparraguirre M , Antonia, Débora Acín Gándara, Eduardo Perea del Pozo, Sandra Dios-Barbeito, Esteban Martin Antona, Manuel Durán Poveda, Peinado Iribar Begon˜ a, Moreno Elola-Olaso Arantxa, Isabel Pascual Migueláñez, Sara Gortázar de las Casas , Gloria Paseiro Crespo, Raul Pardo, David Luengas, Garcia Chiloeches A, Puerta A, Maqueda González R, Gutiérrez Samaniego M, Colao García L, Serrano González J, Núñez O’Sullivan S, Rodriguez Haro C, Vaquero MA, A Picardo Nieto, Vera-Mansilla C, Pérez-González M, Soto Schütte S, Gutiérrez Calvo A, Sanchez Argüeso A, Hernández-Villafranca S, Qian Zhang S, Gorosabel M, Mínguez García J, Casalduero García L, Florez Gamarra M, Arguello Andres JM, Tallon Iglesias B, García Gutierrez V, Pereira Perez F, Aparicio-Sanchez D, Durán-Muñoz-Cruzado V, Pareja-Ciuró F, Cano-Valderrama O, Avellana R, Torres-Garcia AJ, Zarain Obrador L,Garcia Ureña MA, Toribio Vazquez C, Fuenmayor-Valera ML, Michael Pellen, Mohamed Basheer, Afaq Malik, Rhiannon Harries, Kristie Parkins, Naomi Spencer, Zoe Li, Joanna Burridge, Hannah Wynn, Mina Mesri.

Corresponding author: Marina Yiasemidou, PhD [marinayiasemidou@gmail.com](mailto:marinayiasemidou@gmail.com) Hull York Medical School, Hull, UK +44 (0) 7975531067

**Supplementary Materials Index**

| **Authors' contribution** | pag. 3 |
| --- | --- |
| **Supplementary Results** |  |
| Table S1: Characteristics of patient’s with ASIP during 2019 and 2020 | pag. 4 |
| Table S2. Predictors of Surgical treatment in patients treated during 2020 | pag. 5-6 |
| Table S3. Predictors of Laparoscopic approach in patients treated during 2020 | pag. 7-8 |
| Table S4. Morbidity and mortality of ASIP in Spain and the UK | pag. 8 |
| **Comparison between COVID + vs COVID –** |  |
| Table S5. Comparison of COVID-19 positive cases between Spain and UK | pag. 9 |
| Table S6. Characteristics of patient with COVID-19 positive test results | pag. 10 |
| Table S7. Severity of disease in COVID-19 positive patients | pag. 10 |
| Table S8. Surgical treatment in COVID patients: comparison of the number of surgically treated patients between negative patients with positive patients in each country | pag. 10 |
| Table S9. Comparison of the number of severe complications between negative patients with positive patients in each country | pag. 11 |
| Table S10. Comparison of the number of severe complications between negative patients with positive patients in each country | pag. 11 |
| Table S11. Mortality in COVID-19 positive patients | pag. 11 |
| Table S12. Predictors for major complications in 2020 | pag. 12 |

**Authors’ contribution**

**Spain Steering committee:**

Hector Guadalajara, José Luis Muñoz de Nova, Saul Fernandez Gonzalez,

Damian García-Olmo

**UK Steering Committee:**

Marina Yiasemidou, Peter Sedman, FRCS, Sushil Maslekar, David Jayne, Ian Chetter.

In addition to the steering committees, each centre had a regional lead for the purposes of data quality checks, supervision of data collection and obtaining relevant permissions for the research

**Regional Leads Spain:**

Maria Recarte Rico, Luz Divina Juez, Javier García Septiem, Pablo Galindo Jara, Mariana García Virosta, Eduardo Lobo Martínez, Elena Martín-Pérez.

**Regional Leads for UK centres:**

Julian Hance, Richard Egan, Kallingal Riyad, Sonia Lockwood, Andrea Giorga, Annabel Howitt

**Collaborators who conducted data collection**

Fernández-Cebrián José María, Blazquez Martin Agustín, Jover José María. Iparraguirre M , Antonia, Débora Acín Gándara, Eduardo Perea del Pozo, Sandra Dios-Barbeito, Esteban Martin Antona, Manuel Durán Poveda, Peinado Iribar Begon˜ a, Moreno Elola-Olaso Arantxa, Isabel Pascual Migueláñez, Sara Gortázar de las Casas , Gloria Paseiro Crespo, Raul Pardo, David Luengas, Garcia Chiloeches A, Puerta A, Maqueda González R, Gutiérrez Samaniego M, Colao García L, Serrano González J, Núñez O’Sullivan S, Rodriguez Haro C, Vaquero MA, A Picardo Nieto, Vera-Mansilla C, Pérez-González M, Soto Schütte S, Gutiérrez Calvo A, Sanchez Argüeso A, Hernández-Villafranca S, Qian Zhang S, Gorosabel M, Mínguez García J, Casalduero García L, Florez Gamarra M, Arguello Andres JM, Tallon Iglesias B, García Gutierrez V, Pereira Perez F, Aparicio-Sanchez D, Durán-Muñoz-Cruzado V, Pareja-Ciuró F, Cano-Valderrama O, Avellana R, Torres-Garcia AJ, Zarain Obrador L, Garcia Ureña MA, Toribio Vazquez C, Fuenmayor-Valera ML, Michael Pellen, Mohamed Basheer, Afaq Malik, Rhiannon Harries Kristie Parkins, Naomi Spencer, Zoe Li, Joanna Burridge, Hannah Wynn, Mina Mesri.

**Table S1. Characteristics of patient’s with ASIP during 2019 and 2020**

|  | **Spain** | | | | **UK** | | | | **Overall** | | | |
| --- | --- | --- | --- | --- | --- | --- | --- | --- | --- | --- | --- | --- |
|  | *2020* | *2019* | *Total* | *p-value* | *2020* | *2019* | *Total* | *p-value* | *2020* | *2019* | *Total* | *p-value* |
| **Appendicitis** | | | | | | | | | | | | |
| **Mild _nº cases (%)_** | 116 (49.4) | 228 (63.5) | 344 (57.9) | **0.002** | 64 (64.6) | 97 (58.8) | 161 (61.0) | **0.005** | 180 (53.9) | 325 (62.0) | 505 (58.9) | **0.005** |
| **Moderate _nº cases (%)_** | 68 (28.9) | 81 (22.6) | 149 (25.1) |  | 11 (11.1) | 44 (26.7) | 55 (20.8) |  | 79 (23.7) | 125 (23.9) | 204 (23.8) |  |
| **Severe _nº cases (%)_** | 51 (21.7) | 50 (13.9) | 101 (17.0) |  | 24 (24.2) | 24 (14.5) | 48 (18.2) |  | 75 (22.5) | 74 (14.1) | 149 (17.4) |  |
| **Cholecystitis** | | | | | | | | | | | | |
| **Mild _nº cases (%)_** | 46 (34.1) | 73 (42.2) | 119 (38.6) | 0.295 | 47 (48.0) | 114 (57.0) | 161 (54.0) | **0.034** | 93 (39.9) | 187 (50.1) | 280 (46.2) | **0.012** |
| **Moderate _nº cases (%)_** | 71 (52.6) | 82 (48.0) | 154 (50.0) |  | 32 (32.7) | 68 (34.0) | 100 (33.6) |  | 103 (44.2) | 151 (40.5) | 254 (41.9) |  |
| **Severe _nº cases (%)_** | 18 (13.3) | 17 (9.8) | 35 (11.4) |  | 19 (19.4) | 18 (9.0) | 37 (12.4) |  | 37 (15.9) | 35 (9.4) | 72 (11.9) |  |
| **Diverticulitis** | | | | | | | | | | | | |
| **Mild _nº cases (%)_** | 29 (69.0) | 92 (80.7) | 121 (77.6) | **0.001** | 36 (70.6) | 76 (68.5) | 112 (69.1) | 0.878 | 65 (69.9) | 168 (74.7) | 233 (73.3) | **0.022** |
| **Moderate _nº cases (%)_** | 2 (4.8) | 16 (14.0) | 18 (11.5) |  | 8 (15.7) | 21 (18.9) | 29 (17.9) |  | 10 (10.8) | 37 (16.4) | 47 (14.8) |  |
| **Severe _nº cases (%)_** | 11 (26.2) | 6 (5.3) | 17 (10.9) |  | 7 (13.7) | 14 (12.6) | 21 (13.0) |  | 18 (19.4) | 20 (8.9) | 38 (11.9) |  |
| **Perianal Abscess** | | | | | | | | | | | | |
| **Mild _nº cases (%)_** | 88 (83.8) | 149 (85.1) | 237 (84.6) | 0.834 | 42 (93.3) | 54 (91.5) | 96 (92.3) | 0.732 | 130 (86.7) | 203 (86.8) | 333 (86.7) | 0.892 |
| **Moderate _nº cases (%)_** | 13 (12.4) | 18 (10.3) | 31 (11.1) |  | 3 (6.7) | 5 (8.5) | 8 (7.7) |  | 16 (10.7) | 23 (9.8) | 39 (10.2) |  |
| **Severe _nº cases (%)_** | 4 (3.8) | 8 (4.6) | 12 (4.3) |  | 0 (0) | 0 (0) | 0 (0) |  | 4 (2.7) | 8 (3.4) | 12 (3.1) |  |
| **Total** | | | | | | | | | | | | |
| **Mild _nº cases (%)_** | 278 (54.0) | 541 (66.2) | 819 (61.5) | **<0.001** | 188 (64.4) | 341 (63.7) | 529 (64.0) | **0.004** | 466 (57.7) | 882 (65.2) | 1348 (62.4) | **<0.001** |
| **Moderate _nº cases (%)_** | 153 (29.7) | 195 (23.9) | 348 (26.1) |  | 54 (18.5) | 138 (25.8) | 192 (23.2) |  | 207 (25.7) | 333 (24.6) | 540 (25.0) |  |
| **Severe _nº cases (%)_** | 84 (16.3) | 81 (9.9) | 165 (12.4) |  | 50 (17.1) | 56 (10.5) | 106 (12.8) |  | 134 (16.6) | 137 (10.1) | 271 (12.6) |  |

**Table S2. Predictors of Surgical treatment in patients treated during 2020^§^**

|  |  | **No Surgery** | **Surgery** | **p-value** | **OR (IC95%)**** | **p-value** |
| --- | --- | --- | --- | --- | --- | --- |
| **Appendicitis** | | | | | | |
| Age **_median (IQR)_** |  | 50 (32-65) | 38 (26-54) | **0.008** | n.s. |  |
| CCI **_median (IQR)_** |  | 0 (1-3) | 0 (0-1) | **0.006** | n.s. |  |
| Country **_nº cases (%)_** | Spain  UK | 18 (7.5)  33 (33.3) | 221 (92.5)  66 (66.7) | **<0.001** | 0.026 (0.004-0.713) | **<0.001** |
| Delay **_nº cases (%)_** | ≤ 7 days  > 7 days | 22 (8.4)  10 (52.6) | 241 (91.6)  9 (47.4) | **<0.001** | 0.026 (0.004-0.155) | **<0.001** |
| a | Mild  Moderate  Severe | 30 (16.6)  13 (16.7)  7 (9.3) | 151 (83.4)  65 (83.3)  68 (90.7) | 0.299 | Ref  1.591 (0.288-8.805)  11.433 (1.174-111.355) | -  0.595  **0.036** |
| COVID status* **_nº cases (%)_** | Negative  Positive | 26 (14.5)  7 (38.9) | 153 (85.5)  11 (61.1) | **0.016** | 0.100 (0.017-0.583) | **0.010** |
| **Cholecystitis** | | | | | | |
| Age **_median (IQR)_** |  | 68 (52-79) | 58.5 (41-71.2) | **0.002** | n.s. |  |
| CCI **_median (IQR)_** |  | 3 (1-5) | 1 (0-3) | **0.001** | 0.591 (0.362-0.966) | **0.036** |
| Country **_nº cases (%)_** | Spain  UK | 90 (68.7)  90 (90.9) | 41 (31.3)  9 (9.1) | **<0.001** | 0.148 (0.026-0.844) | **0.031** |
| Delay **_nº cases (%)_** | ≤ 7 days  > 7 days | 99 (72.3)  19 (76.0) | 38 (27.7)  6 (24.0) | 0.699 | n.s. |  |
| Severity at diagnosis **_nº cases (%)_** | Mild  Moderate  Severe | 81 (86.5)  71 (71.7)  27 (75.0) | 13 (13.8)  28 (28.3)  9 (25.0) | **0.046** | Ref  2.715 (0.829-8.886)  7.944 (1.357-46.500) | -  0.099  **0.022** |
| COVID status* **_nº cases (%)_** | Negative  Positive | 114 (74.5)  18 (100) | 39 (25.5)  0 (0) | **0.014** | n.s. |  |
| **Diverticulitis** | | | | | | |
| Age **_median (IQR)_** |  | 65 (49-77) | 62.5 (51-74.5) | 0.692 |  |  |
| CCI **_median (IQR)_** |  | 2 (0-4) | 2 (1-3.5) | 0.868 |  |  |
| Country **_nº cases (%)_** | Spain  UK | 32 (69.6)  43 (84.3) | 14 (30.4)  8 (15.7) | 0.083 |  |  |
| Delay **_nº cases (%)_** | ≤ 7 days  > 7 days | 40 (75.5)  7 (63.6) | 13 (24.5)  4 (36.4) | 0.463 |  |  |
| Severity at diagnosis **_nº cases (%)_** | Mild  Moderate  Severe | 57 (91.9)  11 (84.6)  4 (21.1) | 5 (8.1)  2 (15.4)  15 (78.9) | **<0.001** |  |  |
| COVID status* **_nº cases (%)_** | Negative  Positive | 45 (77.6)  5 (71.4) | 13 (22.4)  2 (28.6) | 0.658 |  |  |

§ Excluding perianal abscess due to the low number or non-surgically treated patients

| **Total** | | | | | | |
| --- | --- | --- | --- | --- | --- | --- |
| Age **_median (IQR)_** |  | 65 (46.7-78) | 42 (28-59) | **<0.001** | 0.975 (0.951-0.999) | **0.049** |
| CCI **_median (IQR)_** |  | 2 (0-4) | 0 (0-2) | **<0.001** | 0.663 (0.527-0.833) | **<0.001** |
| Country **_nº cases (%)_** | Spain  UK | 140 (33.7)  166 (66.7) | 276 (66.3)  83 (33.3) | **<0.001** | 0.093 (0.036-0.241) | **<0.001** |
| Delay **_nº cases (%)_** | ≤ 7 days  > 7 days | 161 (35.5)  36 (65.5) | 292 (64.5)  19 (34.5) | **<0.001** | 0.248 (0.104-0.588) | **<0.001** |
| Severity at diagnosis **_nº cases (%)_** | Mild  Moderate  Severe | 168 (49.9)  95 (50.0)  38 (29.2) | 169 (50.1)  95 (50.0)  92 (70.8) | **<0.001** | Ref  1.499 (0.769-2.923)  12.987 (4.794-35.183) | -  0.235  **<0.001** |
| COVID status* **_nº cases (%)_** | Negative  Positive | 185 (47.4)  30 (69.8) | 205 (52.6)  13 (30.2) | **0.005** | 0.142 (0.052-0.389) | **<0.001** |

* Only tested patients

** Total: Logistic regression model

**Table S3. Predictors of Laparoscopic approach in patients treated during 2020^§^**

|  |  | **No Laparoscopic** | **Laparoscopic** | **p-value** | **OR (IC95%)**** | | **p-value** | |
| --- | --- | --- | --- | --- | --- | --- | --- | --- |
| **Appendicitis** | | | | | | | | |
| Age **_median (IQR)_** |  | 41 (25.7-59.2) | 37.5 (27.0) | 0.292 |  | |  | |
| CCI **_median (IQR)_** |  | 0 (0-2) | 0 (0-1) | 0.099 |  | |  | |
| Country **_nº cases (%)_** | Spain  UK | 37 (16.8)  45 (68.2) | 183 (83.2)  21 (31.8) | **<0.001** | 0.093 (0.034-0.253) | | **<0.001** | |
| Delay **_nº cases (%)_** | ≤ 7 days  > 7 days | 65 (27.1)  1 (11.1) | 175 (72.9)  8 (88.9) | 0.452 |  | |  | |
| Severity at diagnosis **_nº cases (%)_** | Mild  Moderate  Severe | 38 (25.3)  17 (26.2)  25 (36.8) | 112 (74.7)  48 (73.8)  43 (63.2) | 0.202 | Ref  0.338 (0.107-1.064)  0.194 (0.064-0.584) | | -  0.064  0.004 | |
| COVID status* **_nº cases (%)_** | Negative  Positive | 29 (19.1)  7 (63.6) | 123 (80.9)  4 (36.4) | **0.003** | 0.126 (0.030-0.520) | | **0.004** | |
| **Cholecystitis** | | | | | |  | |  |
| Age **_median (IQR)_** |  | 50.5 (33.7-70.7) | 58.5 (42.2-71.7) | 0.652 |  | |  | |
| CCI **_median (IQR)_** |  | 1 (1-2.5) | 1 (0-3) | 0.570 |  | |  | |
| Country **_nº cases (%)_** | Spain  UK | 4 (9.8)  2 (22.2) | 37 (90.2)  7 (77.8) | 0.293 |  | |  | |
| Delay **_nº cases (%)_** | ≤ 7 days  > 7 days | 6 (15.8)  0 (0) | 32 (84.2)  6 (100) | 0.573 |  | |  | |
| Severity at diagnosis **_nº cases (%)_** | Mild  Moderate  Severe | 1 (7.7)  3 (10.7)  2 (22.2) | 12 (92.3)  25 (89.3)  7 (77.8) | 0.559 |  | |  | |
| COVID status* **_nº cases (%)_** | Negative  Positive | 4 (10.3)  0 (0) | 35 (89.7)  0 (0) | - |  | |  | |
| **Total** | | | | | | | | |
| Age **_median (IQR)_** |  | 41 (26-60) | 40.5 (28-56) | 0.821 |  | |  | |
| CCI **_median (IQR)_** |  | 0 (0-2) | 0 (0-1) | 0.504 |  | |  | |
| Country **_nº cases (%)_** | Spain  UK | 41 (15.7)  47 (62.7) | 220 (84.3)  28 (37.3) | **<0.001** | 0.152 (0.064-0.360) | | **<0.001** | |
| Delay **_nº cases (%)_** | ≤ 7 days  > 7 days | 71 (25.5)  1 (6.7) | 207 (74.5)  14 (93.3) | 0.128 |  | |  | |
| Severity at diagnosis **_nº cases (%)_** | Mild  Moderate  Severe | 39 (23.9)  20 (21.5)  27 (35.1) | 124 (76.1)  73 (78.5)  50 (64.9) | 0.098 | Ref  0.425 (0.154-1.176)  0.229 (0.085-0.621) | | -  0.099  **0.002** | |
| COVID status* **_nº cases (%)_** | Negative  Positive | 33 (17.3)  7 (63.6) | 158 (82.7)  4 (36.4) | **0.001** | 0.112 (0.028-0.444) | | **0.002** | |

§ Only patients with appendicitis and cholecystitis; excluded diverticulitis due to the small sample size for this item * Only tested patients ** Logistic regression model

**Table S4. Morbidity and mortality of ASIP in Spain and the UK**

|  | **Spain** | | | | **UK** | | | | **Overall** | | | |
| --- | --- | --- | --- | --- | --- | --- | --- | --- | --- | --- | --- | --- |
|  | *2020* | *2019* | *Total* | *p-value* | *2020* | *2019* | *Total* | *p-value* | *2020* | *2019* | *Total* | *p-value* |
| **Major complications _nº cases (%)_** | 37 (7.1) | 45 (5.5) | 82 (6.1) | 0.225 | 16 (5.4) | 15 (2.8) | 31 (3.7) | 0.055 | 53 (6.5) | 60 (4.4) | 113 (5.2) | **0.034** |
| **Mortality _nº cases (%)_** | 5 (1.0) | 7 (0.9) | 12 (0.9) | 1 | 1 (0.3) | 3 (0.6) | 4 (0.5) | 1 | 6 (0.7) | 10 (0.7) | 16 (0.7) | 1 |
| **LOS days _median (IQR)_** | 3 (1-7) | 2 (1-5) | 3 (1-6) | **0.006** | 3 (1-5) | 3 (2-5) | 3 (1-5) | 0.659 | 3 (1-6) | 2 (1-5) | 3 (1-5) | 0.050 |

## **Comparison between COVID + vs COVID – (excluded unknown COVID status)**

**Table S5. Comparison of COVID-19 positive cases between Spain and UK^§^**

|  | **Appendicitis _n (%)_** | **Cholecystitis _n (%)_** | **Diverticulitis _n (%)_** | **Perianal Abscess _n (%)_** | **Total _n (%)_** |
| --- | --- | --- | --- | --- | --- |
| **Spain** | 13 (8.8) | 13 (12.6) | 5 (14.7) | 6 (10) | 37 (10.7) |
| **UK** | 5 (10.2) | 5 (7.4) | 2 (6.5) | 0 (0) | 12 (6.8) |
| **p-value** | 0.777 | 0.272 | 0.430 | 0.171 | 0.149 |

§ COVID +: The comparison is between both countries with the patients of 2020. Patients positive for COVID test between those tested.

**Table S6. Characteristics of patient with COVID-19 positive test results^§^**

|  | **Spain** | | | | **UK** | | | | **Overall** | | | |
| --- | --- | --- | --- | --- | --- | --- | --- | --- | --- | --- | --- | --- |
|  | *(-)* | *(+)* | *Total* | *p-value* | *(-)* | *(+)* | *Total* | *p-value* | *(-)* | *(+)* | *Total* | *p-value* |
| **Age**  **_median (IQR)_** | 51.5  (35-70) | 54  (35-75.5) | 52  (35-71.5) | 0.407 | 53  (34.2-70) | 73  (60.5-78.5) | 54.5  (36.2-71) | **0.002** | 52  (35-70) | 59  (43.5-77) | 53  (35-71) | **0.028** |
| **Male _nº cases (%)_** | 192 (62.5) | 21 (56.8) | 213 (61.9) | 0.494 | 81 (49.4) | 6 (50.0) | 87 (49.4) | 0.967 | 273 (58.0) | 27 (55.1) | 300 (57.7) | 0.700 |
| **CCI _median (IQR)_** | 1 (0-3) | 1 (0-5) | 0 (1-4) | 0.289 | 1 (0-3) | 4 (2-5.7) | 1 (0-3) | **<0.001** | 1 (0-3) | 2 (0-5) | 1 (0-3) | **0.007** |
| **Delay > 3 d**  **_nº cases (%)_** | 153 (49.7) | 23 (62.2) | 176 (51.0) | 0.151 | 18 (39.1) | 2 (50.0) | 20 (40.0) | 1 | 171 (48.3) | 25 (61.0) | 196 (49.6) | 0.125 |

§Only tested patients

**Table S7**. **Severity of disease in COVID-19 positive patients ^§^**

|  | **Appendicitis _n (%)_** | | | | **Cholecystitis _n (%)_** | | | | **Diverticulitis _n (%)_** | | | | **Perianal Abscess _n (%)_** | | | | **Total _n (%)_** | | |
| --- | --- | --- | --- | --- | --- | --- | --- | --- | --- | --- | --- | --- | --- | --- | --- | --- | --- | --- | --- |
|  | **Spain** | **UK** | **Total** | **Spain** | | **UK** | **Total** | **Spain** | | **UK** | **Total** | **Spain** | | **UK** | **Total** | **Spain** | | **UK** | **Total** |
| **Negative** | 24 (18) | 12 (27.3) | 36  (20.3) | 11 (12.2) | | 11 (17.7) | 22 (14.5) | 7  (25) | | 3 (11.1) | 10  (18.2) | 3  (5.6) | | 0  (0) | 3  (3.7) | 45  (14.8) | | 26  (16.3) | 71  (15.3) |
| **Positive** | 5 (38.5) | 2  (40) | 7  (38.9) | 4 (30.8) | | 3  (60.0) | 7 (38.9) | 2  (40) | | 0  (0) | 2 (28.6) | 0  (0) | | 0  (0) | 0  (0) | 11  (29.7) | | 5  (41.7) | 16  (32.7) |
| **p-value** | 0.136 | 0.616 | 0.080 | 0.094 | | 0.058 | **0.017** | 0.597 | | 1 | 0.612 | 1 | | - | 1 | **0.020** | | **0.043** | **0.002** |

§Comparison of number of severe cases between negative patients with positive patients in each country (only tested patients).

**Table S8. Surgical treatment in COVID patients: comparison of the number of surgically treated patients between negative patients with positive patients in each country^§^**

|  | **Appendicitis _n (%)_** | | | **Cholecystitis _n (%)_** | | | | **Diverticulitis _n (%)_** | | | | **Perianal Abscess _n (%)_** | | | | **Total _n (%)_** | | |
| --- | --- | --- | --- | --- | --- | --- | --- | --- | --- | --- | --- | --- | --- | --- | --- | --- | --- | --- |
|  | **Spain** | **UK** | **Total** | **Spain** | **UK** | **Total** | **Spain** | | **UK** | **Total** | **Spain** | | **UK** | **Total** | **Spain** | | **UK** | **Total** |
| **Negative** | 125  (92.6) | 28  (63.6) | 153  (85.5) | 31  (34.4) | 8  (12.7) | 39  (25.5) | 10  (34.5) | | 3  (10.3) | 13  (22.4) | 53  (98.1) | | 27  (96.4) | 80  (97.6) | 219  (71.1) | | 66  (40.2) | 285  (60.4) |
| **Positive** | 9  (69.2) | 2  (40) | 11  (61.1) | 0  (0) | 0  (0) | 0  (0) | 2  (40) | | 0  (0) | 2  (28.6) | 6  (100) | | - | 6  (100) | 17  (45.9) | | 2  (15.4) | 19  (38) |
| **p-value** | **0.022** | 0.363 | **0.016** | **0.009** | 1 | **0.008** | 1 | | 1 | 0.658 | 1 | | - | 1 | **0.002** | | 0.136 | **0.002** |

§Only tested patients

|  | **Appendicitis _n (%)_** | | | **Cholecystitis _n (%)_** | | | **Diverticulitis _n (%)_** | | | | **Perianal Abscess _n (%)_** | | | | **Total _n (%)_** | | |
| --- | --- | --- | --- | --- | --- | --- | --- | --- | --- | --- | --- | --- | --- | --- | --- | --- | --- |
|  | **Spain** | **UK** | **Total** | **Spain** | **UK** | **Total** | **Spain** | **UK** | **Total** | **Spain** | | **UK** | **Total** | **Spain** | | **UK** | **Total** |
| **Negative** | 8  (5.9) | 2  (4.5) | 10  (5.6) | 6  (6.7) | 2  (3.2) | 8  (5.2) | 6  (20.7) | 2  (6.9) | 8  (13.8) | 2  (3.7) | | 5  (17.9) | 7  (8.5) | 22  (7.1) | | 11  (6.7) | 33  (7) |
| **Positive** | 1  (7.1) | 0  (0) | 1  (5.6) | 4  (30.8) | 1  (20.0) | 5  (27.8) | 1  (20) | 0  (0) | 1  (14.3) | 1  (16.7) | | - | 1  (16.7) | 7  (18.9) | | 1  (8.3) | 8  (16.3) |
| **p-value** | 0.573 | 1 | 1 | **0.021** | 0.208 | **0.006** | 1 | 1 | 1 | 0.275 | | - | 0.446 | **0.024** | | 0.584 | **0.044** |

**Table S9. Comparison of the number of severe complications between negative patients with positive patients in each country**^§^

§Only tested patients

**Table S10. Comparison of the number of severe complications between negative patients with positive patients in each country**^§^

|  | **Appendicitis _n (%)_** | | | **Cholecystitis _n (%)_** | | | **Diverticulitis _n (%)_** | | | **Perianal Abscess _n (%)_** | | | **Total _n (%)_** | | |
| --- | --- | --- | --- | --- | --- | --- | --- | --- | --- | --- | --- | --- | --- | --- | --- |
|  | **Spain** | **UK** | **Total** | **Spain** | **UK** | **Total** | **Spain** | **UK** | **Total** | **Spain** | **UK** | **Total** | **Spain** | **UK** | **Total** |
| **Negative** | 8  (6.4) | 2  (7.1) | 10  (6.5) | 2  (6.5) | 1  (12.5) | 3  (7.7) | 5  (50) | 1  (33.3) | 7  (56.2) | 2  (3.8) | 4  (14.8) | 6  (7.5) | 17  (7.8) | 8  (12.1) | 25  (8.8) |
| **Positive** | 1  (11.1) | 0  (0) | 1  (9.1) | - | - | - | 1  (50) | - | 1  (50) | 1  (16.7) | - | 1  (16.7) | 3  (17.6) | 0  (0) | 3  (15.8) |
| **p-value** | 0.476 | 1 | 0.546 | - | - | - | 1 | - | 1 | 0.279 | - | 0.409 | 0.164 | 1 | 0.400 |

§Only tested patients

**Table S11. Mortality in COVID-19 positive patients**

|  | **Appendicitis _n (%)_** | | | **Cholecystitis _n (%)_** | | | **Diverticulitis _n (%)_** | | | **Perianal Abscess _n (%)_** | | | **Total _n (%)_** | | |
| --- | --- | --- | --- | --- | --- | --- | --- | --- | --- | --- | --- | --- | --- | --- | --- |
|  | **Spain** | **UK** | **Total** | **Spain** | **UK** | **Total** | **Spain** | **UK** | **Total** | **Spain** | **UK** | **Total** | **Spain** | **UK** | **Total** |
| **Negative** | 0  (0) | 0  (0) | 0  (0) | 3  (3.3) | 0  (0) | 3  (2) | 0  (0) | 0  (0) | 0  (0) | 0  (0) | 0  (0) | 0  (0) | 3  (1) | 0  (0) | 3  (0.6) |
| **Positive** | 0  (0) | 0  (0) | 0  (0) | 2  (15.4) | 0  (0) | 2  (11.1) | 0  (0) | 0  (0) | 0  (0) | 0  (0) | 0  (0) | 0  (0) | 2  (5.4) | 0  (0) | 2  (4.1) |
| **p-value** | - | - | - | 0.119 | - | 0.087 | - | - | - | - | - | - | 0.091 | - | 0.072 |

**Table S12. Predictors for major complications in 2020**

|  |  | **Clavien 0-2** | **Clavien 3-5** | **p-value** | **OR (IC95%)**** | **p-value** |
| --- | --- | --- | --- | --- | --- | --- |
| Age**_median (IQR)_** |  | 50 (34-67) | 61 (35.5-77) | 0.110 |  |  |
| CCI**_median (IQR)_** |  | 1 (0-3) | 2 (0-4) | 0.072 | 1.084 (0.956-1.230) | 0.209 |
| Country **_nº cases (%)_** | Spain  UK | 484 (92.9)  281 (94.6) | 37 (7.1)  16 (5.4) | 0.338 |  |  |
| Surgical treatment **_nº cases (%)_** | No  Yes | 292 (94.8)  472 (92.7) | 16 (5.2)  37 (7.3) | 0.243 |  |  |
| Severity at diagnosis **_nº cases (%)_** | Non-severe  Severe | 640 (95.1)  114 (85.1) | 33 (4.9)  20 (14.9) | **<0.001** | **3.148 (1.487-6-663)** | **0.003** |
| COVID status* **_nº cases (%)_** | Negative  Positive | 439 (93.0)  41 (83.7) | 33 (7.0)  8 (16.3) | **0.044** | 1.717 (0.672-4.384) | 0.259 |
